# Suicide after reception into prison: A case-control study examining differences in early and late events

**DOI:** 10.1371/journal.pone.0255284

**Published:** 2021-08-03

**Authors:** Daniel Radeloff, Marian ten Hövel, Gerald Brennecke, Franziska S. Stoeber, Thomas Lempp, Mattias Kettner, Hannes Zacher, Kai von Klitzing, Katharina Bennefeld-Kersten

**Affiliations:** 1 Department of Child and Adolescent Psychiatry, Psychotherapy and Psychosomatics, University Hospital Leipzig, Leipzig, Germany; 2 Chair for Work and Organizational Psychology, Institute of Psychology, Leipzig University, Leipzig, Germany; 3 Clementine Children’s Hospital, Frankfurt/Main, Germany; 4 Institute of Forensic Medicine, Goethe University Frankfurt, Frankfurt, Germany; 5 Institute for Suicide Research, Restorf-Hohbeck, Germany; University of New South Wales, AUSTRALIA

## Abstract

**Objective:**

Prisoners constitute a high-risk group for suicide, with suicide rates about 5 to 8 times higher than in the general population. The first weeks of imprisonment are a particularly vulnerable time, but there is limited knowledge about the risk factors for either early or late suicide events.

**Methods:**

Based on a national total sample of prison suicides in Germany between 2005 and 2017, suicides within the first 2 (4 and 8) weeks after reception into prison were matched by age and penalty length with cases that occurred later. Factors that potentially influence the timing of suicide were investigated.

**Results:**

The study has shown that 16.7% (31.5%) of all 390 suicides in German prisons occurred within the first two weeks (two months) of imprisonment. Factors that facilitate adaptation to the prison environment (e.g. prior prison experience) were negatively associated with early suicide events. Factors that hindered the adaptation process (e.g. withdrawal from illicit drugs) were observed more frequently in early suicide events than in late ones. These factors are active at different times of imprisonment.

**Conclusion:**

At reception, particular attention should be paid to the following factors associated with early suicide events: widowed marital status, lack of prison experience, and drug dependency.

## Introduction

Suicide is a major challenge for the public health system, accounting for over 800,000 deaths each year worldwide [1]. In Germany, as in many Western countries, suicide is the most prevalent non-natural cause of death [2]. According to WHO guidelines, it is crucial to identify risk populations and to provide them with psychiatric support [3].

Prisoners constitute such a risk group. Several large epidemiological studies have shown that delinquency is associated with suicide [4–7]. Prisoners represent one extreme on the spectrum of delinquency, and are exposed to a particularly high risk of suicide, with suicide rates about 5 to 8 times higher than in the general population [8,9]. There are two reasons for this. First, prisoners show behaviour and personality traits associated with suicide, even before imprisonment; these risk factors are imported into the prison environment. For example, prisoners show high rates of suicide attempts, impulsiveness, mental disorders, and drug abuse [10–12]. Second, this highly vulnerable population is exposed to further stressors during imprisonment, such as loss of control, violence among prisoners, social disapproval, separation from family and friends, and dealing with feelings of guilt and shame [13–15].

Suicide risk is unequally distributed among prisoners. Adolescent prisoners are exposed to a particularly high relative risk of suicide [8,16]. Suicide rates in remand detention are considerably higher than following conviction [9,17,18]. Prisoners convicted of violent offences, such as offences against life, sexual offences, or bodily harm are exposed to a particularly high suicide risk [17,19]. Temporal and situational risk factors have also been described: for example, the time preceding the pronouncement of judgement, weekdays with low staff density, and anniversaries of imprisonment have all been identified as risk periods [20–23].

The first weeks of imprisonment are particularly important for suicide prevention, since a considerable proportion of suicides in prisons occur during this period [20,23,24]. Admission to a prison represents a major change in a person’s life situation, and requires a considerable effort to adapt. The more abrupt and serious such a change is, the greater this effort has to be. A sudden admission to pre-trial detention may be more incisive than the anticipated entry into prison after sentencing. Stressors are those individual, criminological, medical and environmental factors that may contribute to increased pressure to adapt (for example, drug addiction and withdrawal, single-cell accommodation, experience of violence in the prison, separation from familiar people providing support) [20,21,25–27]. Protective factors are those that facilitate this adaptation process, such as previous prison experience, a transparent introduction to prison procedures, or the availability of psychological support.

Only isolated studies have examined whether these factors are related to early suicide in prison [28,29], and these show a connection between drug addiction and early prison suicide.

To the best of our knowledge, this is the first study that uses a case-control design to investigate whether suicides in the first weeks of imprisonment differ from late prison suicide events in terms of their risk and resilience factors.

This study aims to address the following hypotheses:

- Previous prison sentences are negatively associated with early suicides, as this knowledge of the prison environment facilitates the process of re-adaptation.
- Having a partner or being married is a protective factor regarding early suicide events.
- Offences that are closely associated with drug use, such as theft, or offences against the narcotics law, are associated with early suicide events.
- The risk of early prison suicides is lower if the convicted person has been informed of the date the sentence will commence. The same applies if the offender turns himself in.
- Evidence of mental illness or drug withdrawal is associated with early prison suicides.
- Assignment of a psychiatrist is protective against suicides during the first days of detention.
- Risk factors change with increasing prison time.

## Methods

### Sample

Data were analysed using IBM SPSS 25.0 [27], Microsoft Excel, and the R software version 3.3.1 [30], with the extension packages “haven” and “e1071”. The dataset was analysed retrospectively using a case-control approach.

The dataset analysed comprises N = 869 suicides, 44.9% (N = 390) of which were carried out after sentencing, 48.4% (N = 421) on remand, and 6.7% (N = 58) in other facilities, such as high-security psychiatric hospitals, or detention pending deportation. Descriptive statistics were calculated in this dataset of prisoner suicides (proportions of suicide within 14, 28, 60, and >60 days of imprisonment). Only suicides in criminal custody were included in the study, as in these cases a court judgement had confirmed an index offence, and set a penalty length. Since approximately 95% of the prison population are male [27], the 15 female cases were excluded. To avoid outliers, penalty length was limited to 10 years, resulting in 36 excluded cases. Three cases were excluded because data were missing on penalty length or index crime. Thirty cases were excluded for lack of secondary data. In total, 291 were qualified for further matching.

#### Matching procedures and analytical strategy

In this study, three time points were examined: 14 days, 28 days and 60 days after entering imprisonment. For each time point, the group of suicides was divided, pairing early suicide events (G_E_) with a group of late suicide events (G_L_), resulting in three pairs (G_E14_/G_L14_; G_E28_/G_L28_; G_E60_/G_E60_).

We then matched each pair according to the variable penalty length, to avoid offences with high penalties under the penal code being over-represented in G_E_ and under-represented in G_L_.

Since suicide rates increase with age, groups were also matched by age. Matching procedures were performed using R (Version 3.3.1). For the pairs G_E14_/G_L14_ and G_E28_/G_L28_, 1:2 matching was performed. For the pair G_E60_/G_L60_, 1:1 matching was performed. 1:2 matching was not successful because of the group strength of G_E60_.

**Fig 1:**
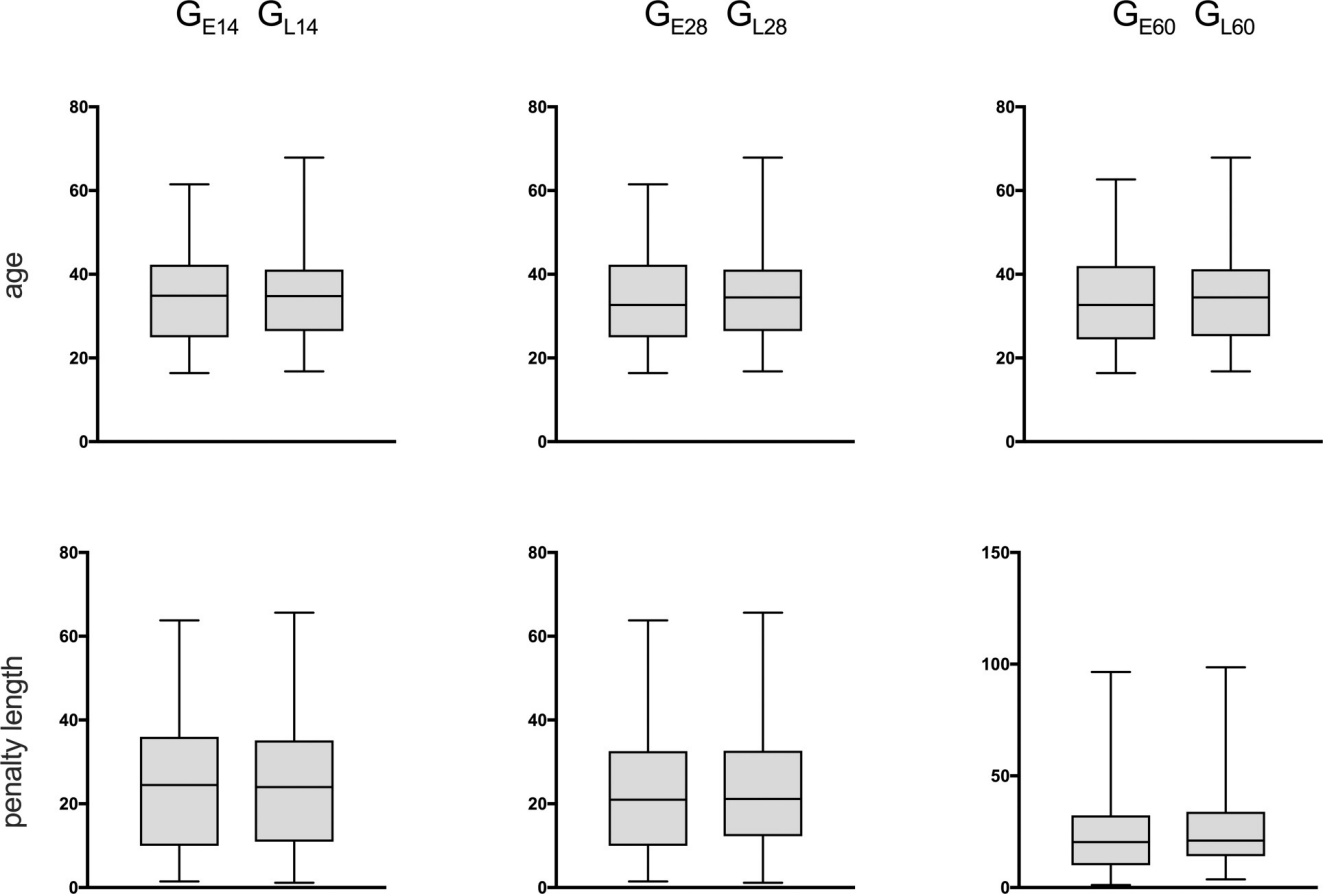
Characteristics of the matching procedure. For each time point examined (14 days, 28 days, and 60 days after entering imprisonment), a group of early (GE) and late suicide (GL) events were matched according to age and penalty length, resulting in three pairs (GE14/GL14; GE28/GL28; GE60/GE60). For each group, box and whisker graphs are given, representing minimum and maximum values, 25th/75th percentiles, and mean values.

*χ^2^*-tests for independent samples were applied to examine whether the hypothesis-specific subsamples of G_E_ and G_L_ with missing values differed on average in the matching variables of age and penalty length. A t-test for independent samples showed that the groups did not differ significantly after matching in age (G_E14/L14:_ t(175) = -.108, p = .914, d = .05; G_E28/L28:_ t(211) = -.085, p = .932, d = .05; G_E60/L60:_ t(208) = -0.172, p = .864, d = .05) or penalty length (G_E14/L14:_ t(175) = -.143, p = .886; d = .05; G_E28/L28:_ t(211) = -.231, p = .932; d = .05; G_E60/L60:_ t(208) = -.706, p = .481, d = .05). For details, see Fig 1.

After matching, *χ^2^*-tests were used to examine associations between the potential influences of mental health, and criminological, educational and social factors, and the allocation to G_E_ or G_L_. Fisher’s exact test was used in crosstables with low cell occupancy. For an overview of the factors examined, see Table 1. Significance level was set at p < .05; results at p<0.1 were considered to be a trend. An overview of factors examined is presented in Table 1.

**Table 1:** Overview of factors with potential influence on the timing of prison suicide.

parameter	values
**Mental health factors**	
Evidence of…	
mental disorder	yes/no
withdrawal of illicit drugs	yes/no
withdrawal of alcohol	yes/no
**Criminological factors**	
previous imprisonment	never/once/multiple/missing
deferment of sentence	yes—and prisoner did report für custodial sentence after summons/yes—and prisoner did not report/no

Index offence	
offences against life	life/other offence
sexual offences	sex offence/other offence
robbery	robbery/other offence
bodily injury	bodily injury/other offence
theft	theft/other offence
fraud	fraud/other offence
narcotics	narcotics/other offence
**Educational factors and social life**	
relationship status	single or living alone/married or partnered/widowed
school qualification	none/special education school/lower secondary qualification/higher secondary qualification/high school graduation


#### Definition of index offence and deferment of sentence

The majority of prisoners have been convicted of multiple offences. An index offence was therefore defined as the offence with the highest severity of legal penalty of the current sentence. Offences were classified into one of eight groups: (1) offences against life, e.g. homicide, (2) offences against sexual self-determination, e.g. sexual abuse, rape, (3) offences against physical integrity, e.g. grievous bodily harm, (4) robbery and extortion, (5) theft, (6) fraud, (7) offences related to violations of the narcotics law, and (8) other offences. In Germany, a sentence does not have to commence immediately. A deferment of up to 4 months can be granted for various reasons, such as to receive urgent medical treatment, to complete one’s education, to settle financial or professional matters, or for lack of a suitable prison place. A person who does not report for a custodial sentence, despite having been summoned to do so, may be subject to an arrest warrant.

#### Ethical considerations

Data had been collected by the prison as part of its operation. Anonymized data on the individual suicide cases were requested via a questionnaire by KBK. These data were made available to the authors (DR, GB) for further analysis. No consent was necessary or possible for this procedure.

The ethics committee did not raise any concerns about this procedure. The study was approved by the ethics committee of the medical faculty of the University Hospital Leipzig, Germany, and conducted according to the Declaration of Helsinki.

## Results

**Fig 2:**
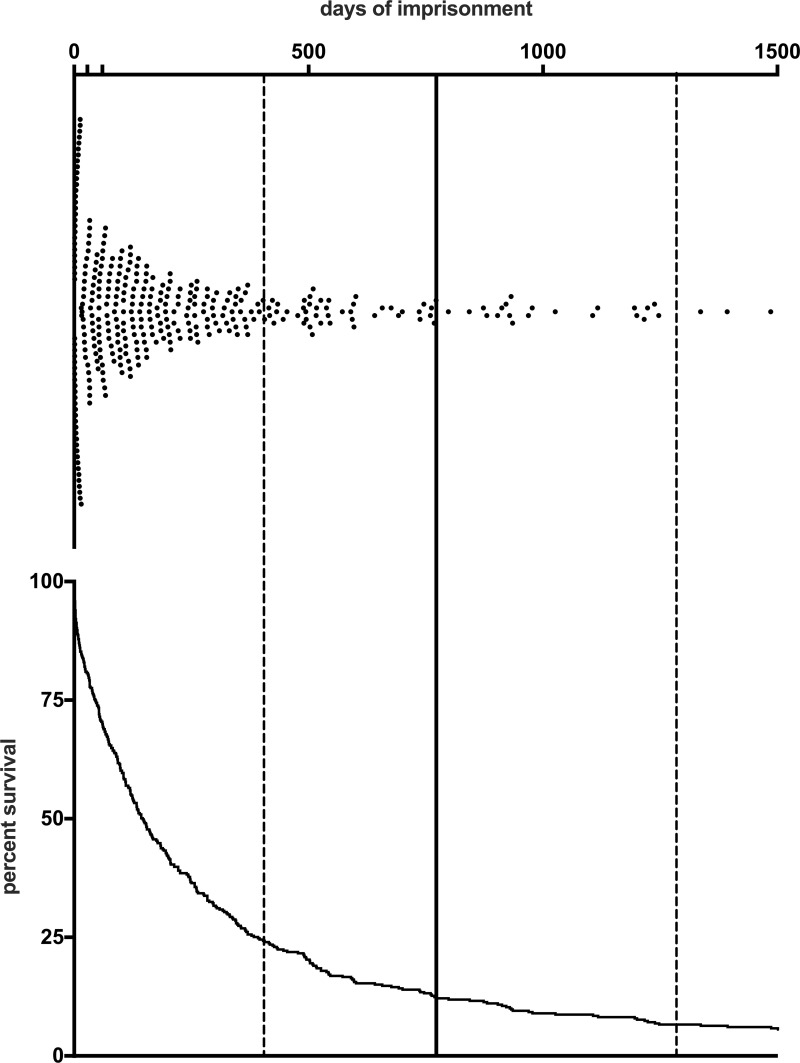
Timing of suicide events. Scatterplot of all suicide events that occurred within the first 1500 days of imprisonment (above) and the corresponding survival curve (below). On the X-axis, additional ticks indicate days 28 and 60 of imprisonment. Vertical lines mark the median (bold line) and the 25th/75th percentile of penalty length (dotted lines).

Of the total number of 390 suicides in prison, the median of the time of suicide was 134 days, and the interquartile range was 332 days. Ten percent of suicides occurred within the first 3 days after reception into prison, 25% of suicides within the first 33 days in prison, and 75% within the first 360 days. Suicides occurred within the first 14, 28 and 60 days of imprisonment, N = 65 (16.7%), N = 82 (21.0%) and N = 123 (31.5%), respectively. The median of the total penalty length was 770 days (P25 = 404 days, P75 = 1285 days). For details, see Fig 2.

The matching process assigned N = 59 suicides in group G_E14_ to N = 128 suicides in group G_L14_ (1:2 matching), and N = 71 suicides in group G_E28_ to N = 142 suicides in group G_L28_ (1:2 matching). In G_60_, 1:2 matching did not successfully generate groups with equal distributions of age and penalty length. Thus, 1:1 matching was applied and N = 105 suicides in group G_E60_ were assigned to N = 105 suicides in group G_L60_.

The associations between risk factors investigated and the time point of suicide are shown for the respective groups in Table 2. The analysis of G_14_ showed that the involvement of a psychiatrist was negatively associated with early prison suicides (*χ^2^* = 11.609, p = 0.001). Relationship status was associated with the timing of suicide: a post hoc analysis showed that being widowed was positively associated with early prison suicide (Fisher’s exact: p = 0.034), whereas being single or partnered had no influence on the timing. The experience of multiple previous episodes of imprisonment was negatively associated with early prison suicide (*χ^2^* = 6.898, p = 0.009). As a trend, a positive association between illicit drug use and early prison suicide was found (*χ^2^* = 2.905, p = 0.088).

**Table 2:** Associations between mental health, criminological, and social factors on timing of prison suicides.

parameter	14 days					28 days					60 days				
	valid data	df	P	χ^2^		valid data	df	P	χ^2^		valid data	df	P	χ^2^	
**Mental health factors**															
Evidence of…															
mental disorder	175/177	1	.104	2.641		211/213	1	.086	2.947	○	209/210	1	.100	2.713	○
withdrawal of illicit drugs	175/177	1	.088	2.905	○	211/213	1	.008	7.143	●	209/210	1	.003	8.777	●
withdrawal of alcohol	175/177	1	.988	0.000		211/213	1	.426	0.634		209/210	1	.410	0.678	
**Criminological factors**															
previous imprisonment	166/177	2	.020	7.827	●	201/213	2	.143	3.883		199/210	2	.224	2.996	
deferment of sentence	138/177	2	.276	2.573		163/213	2	.090	4.823	○	158/210	2	.077	5.127	○
Index offence															
offences against life	177/177	1	.585	0.297		213/213	1	.915	0.011		210/210	1	.552	0.354	
sexual offences	177/177	1	.252	1.311		213/213	1	.406	0.690		210/210	1	.011	6.402	●
robbery	177/177	1	.737	0.113		213/213	1	.066	3.381	○	210/210	1	.206	1.600	
bodily injury	177/177	1	.801	0.063		213/213	1	.207	1.590		210/210	1	.868	0.027	
theft	177/177	1	.904	0.015		213/213	1	.915	0.011		210/210	1	.359	0.840	
fraud	177/177	1	.598	0.278		213/213	1	.406	0.690		210/210	1	.810	0.058	
narcotics	177/177	1	.170	1.887		213/213	1	.066	3.381	○	210/210	1	.367	0.812	
**Educational factors and social life**															
relationship status	175/177	2	.042	6.333	●	211/213	2	.044	6.263	●	114/210	2	.209	3.133	
school qualification	97/177	4	.830	1.479		120/213	4	.559	2.994		208/210	4	.560	2.989	

Column 14 days (28 days, 60 days) shows the results of the group comparison GE14/GL14 (GE28/GL28, GE60/GL60). Significant results are marked with a black dot, trends with a white dot.

Analysis of G_28_ showed that evidence of withdrawal from illicit drugs was positively associated with early suicides (*χ^2^* = 7.143, p = 0.008), but the involvement of a psychiatrist was negatively associated with early suicide events (*χ^2^* = 10.686, p = 0.001).

Again, a post hoc analysis of relationship status showed that being widowed was positively associated with early prison suicides (Fisher’s exact: p = 0.034), whereas being single or partnered had no influence on the timing. As trends, positive associations were found between early suicides and evidence of mental disorders (*χ^2^* = 2.947, p = 0.086), being convicted for robbery (*χ^2^* = 3.381, p = 0.066), or drug offences (*χ^2^* = 3.381, p = 0.066). Some prisoners are given a deferment of sentence and others enter custody abruptly. This has an impact on the timing of the suicide (df = 2, *χ^2^* = 3.381, p = 0.066). A post hoc analysis of the factor “deferment of sentence” showed that prisoners who, despite being convicted, did not appear for their sentence, were at greater risk of suicide in the early stages of their sentence (df = 1, *χ^2^* = 4.115, p = 0.043).

The analysis of G_60_ revealed that early suicide was positively associated with withdrawal from illicit drugs (df = 1, *χ^2^* = 8.777, p = 0.003), negatively associated with the involvement of a psychiatrist (df = 1, *χ^2^* = 12.831, p < 0.001), and negatively associated with sexual offences (df = 1, *χ^2^* = 6.402, p = 0.011). Trends of negative associations were found between early suicide and evidence of mental disorders (df = 1, *χ^2^* = 2.713, p = 0.100) and “deferment of sentence” (df = 2, *χ^2^* = 5.127, p = 0.077). A post hoc analysis of “deferment of sentence” showed that prisoners who failed to appear for their sentence were at more risk of suicide in the early stages of this sentence (df = 1, χ^2^ = 3.076, p = 0.080), whereas those who did respond to the summons were at lower risk of suicide in the early stages of their sentence (df = 1, χ^2^ = 3.712, p = 0.054).

## Discussion

The study yielded the following main results. Early and late suicides in prison are associated with different risk and resilience factors. Factors that facilitate adaptation to the prison environment, such as multiple previous prison sentences, showed a negative association with early suicide events. On the other hand, factors that make this adaptation process more difficult were observed more frequently in cases of early prison suicide events than in late ones. These factors include evidence of withdrawal from illegal drugs or widowed marital status. Surprisingly, sexual offences were associated with late prison suicides, although it can be assumed that these offences are accompanied by a high degree of adaptation in the prison peer group. While some influencing factors (prior prison experience, marital status) take effect mainly in the first two to four weeks of imprisonment, others (indication of withdrawal symptoms) take effect between 4 and 12 weeks into a sentence.

There is a multilevel approach to suicide prevention in prisons, including key components like training and awareness programmes for the prison staff, intake screening for and ongoing observation of suicidal intent, improvement of architecture, and management following screening, i.e. mental health treatment [20,31,32].

A substantial proportion of all suicides in prisons occurs in the first days of imprisonment [20,23,24,33]. The present study has shown that between 2005 and 2017, 13.2% of all 390 suicides in German prisons took place within the first two weeks of imprisonment, and 31.3% within the first two months (Fig 2). This proportion of early suicides was lower than other studies have reported [28,29,34,35]. These differences can be explained by the fact that the study only considered suicides after sentencing, while excluding those on remand. Remand status increases the risk of suicide within the first seven days of detention [23,28].

The very high proportion of early suicide events in prison underlines the need for early prevention strategies, and the chances they present. The WHO therefore recommends an initial screening for suicide risk factors within the first 24 hours of imprisonment [31]. There is a growing evidence base for risk factors for prison suicides in general, including criminological, mental health, social and environmental factors [16,18,19,36]. Below, we discuss the individual protective and risk factors associated with the time frame of early prison suicides.

### Criminological factors

Prison suicide is associated with criminological risk factors, such as being convicted of violent offences (homicide, sexual offences, bodily injury), serving a life sentence, or being on remand [16,18,32,36,37]. Large-scale epidemiological studies have shown an association between suicide risk in the general population and the frequency of convictions in delinquent minors and adults [4,6]. In prison populations, prior convictions were not clearly associated with suicide risk [36]. These studies did not evaluate the influence of criminological factors on the timing of prison suicides.

In our study, prior experience of prison (two or more previous prison sentences) was negatively associated with suicide within the first 14 days of imprisonment. This is in line with hypothesis A, since adaptation to the prison environment may be easier when the general framework is known. Individuals without prior experience of imprisonment, as well as those with a single previous prison sentence, were more vulnerable to suicide within the first 14 days. It seems essential to inform individuals without prison experience about the procedures, contact persons and assistance in prison, and to assign a prisoner who is familiar with the routines as peer support [20].

According to German law, persons who have been convicted can request a deferment of sentence and will later be summoned to enter custody. Convicted indiviuals who had received a summons to begin a prison sentence but did not appear for it showed an increased risk of suicide in the early phase of imprisonment. This is in line with hypothesis D. We can speculate that these prisoners may not be facing up to their changed living conditions, but are trying to avoid them. The effort required to adapt is greater if they have not already internally confronted and recognised this changed reality.

Contrary to hypothesis C, theft and offences against the narcotics law were not clearly linked to early suicide events. Only at one of the time points investigated was a trending association found between early suicide events and drug offences. While our study did not find a clear association between drug offences and early suicide events, there was an association between withdrawal symptoms and early prison suicide, which will be discussed later. The WHO guideline on suicide prevention in prison recommends taking a history of addiction on the day of admission, and checking for acute intoxication [31]. A negative association was found between sexual offences and early suicides. This finding is unclear and requires further investigation.

### Social contact and marital status

Social inclusion is considered a protective factor with regard to suicide in general [38]. Based on three original studies [39–41], a recent meta-analysis [36] reported a doubled suicide risk when prisoners were not visited. This is in line with the findings of studies investigating the association between near-lethal self-harm in prisons and social support [42]. While detention considerably limits social contacts with familiar persons, these findings illustrate that social contacts are essential for prisoners. Although being married is considered a protective factor for suicide in the general population [43], suicide risk in prisoners is higher when they are married [36]. This underscores that separation from partners and families is a stressor for prisoners, and it may thus represent a risk factor for suicide.

The present study, however, found no differences regarding the timing of prison suicide events between prisoners with or without a partner. This contrasts with hypothesis B. However, we did find a higher proportion of widowed individuals among the suicides in the early stages of imprisonment. Widowed status may be due to intimate partner killings, which are associated with loss of control, jealousy, and relationship termination, often carried out in the heat of the moment [44]. Accordingly, these prisoners may be characterised by a higher degree of impulsivity and may be confronted with intense feelings of guilt during the subsequent weeks of detention. Prisoners who are widowed as a result of their offence must therefore be considered particularly vulnerable to early suicide events. However, data are based on only a few widowed individuals, all of whom died in the early stages of imprisonment.

### Mental disorders

An essential role of suicide prevention is the identification of mental disorders and their prompt and competent treatment [45]. This applies in particular to suicide prevention among prisoners [31], since mental disorders, including affective disorders, ADHD, personality disorders, alcohol and drug abuse, prior maltreatment, and suicide attempts are highly prevalent in this population [32,46–50]. Even before intake, prisoners show a high prevalence of previous suicide attempts or drug withdrawal treatment [51]. This highly volatile population may be particularly challenged in adapting to the prison environment. In our study, drug withdrawal symptoms, for example, were associated with suicide in the first 4 to 12 weeks of imprisonment (hypothesis E). This finding replicates an association between drug dependence and suicide within 7 days after reception into prison reported in a study examining 688 suicides during remand or sentence in England and Wales [28]. A second study found that early suicides were more common in drug-dependent prisoners: 22 of these (59%) died within 7 days of reception [29]. In about a quarter of the suicides in Berlin prisons, toxicological analyses showed the use of drugs, while drug overdose was rarely used as a suicide method [52].

As a trend, mental disorders are associated with early suicide events in prison (hypothesis E), while psychiatric care is negatively associated with early suicide events (hypothesis F). All this underlines the fact that local psychiatric expertise is an essential component in the prevention of early prison suicide. Mentally ill people must receive full support at the beginning of their imprisonment, which includes guidance in the adaptation process during their first weeks of detention.

This study demonstrated that the factors influencing suicide events change with the length of imprisonment (hypothesis G). While marital status and prison experience are particularly relevant during the first 14 days as factors influencing the timing of suicide events, they recede in favour of mental health factors in the further course of imprisonment (Table 2). Psychiatric support seems to be consistently relevant.

## Conclusion

From the results of this study, we conclude that the following prisoners are at increased risk of suicide early in imprisonment: (1) widowed prisoners, especially if they have killed their partner, (2) individuals without any prison experience, (3) patients with a drug addiction or psychiatric illness, especially those receiving no psychiatric treatment, and (4) individuals who failed to turn up for prison despite being sentenced. As a prevention strategy, a concept for supporting adaptation processes in prisons should be established and evaluated. The influencing factors mentioned may help to identify particularly challenged individuals. Studies with a prospective approach, but also meta-analytical evaluations of timing of suicide events are necessary to counter early suicide in prison in the future effectively.

## Limitations and strengths

This study used an exact matching approach to examine early and late suicide events in prison in a differentiated way, which is essential, since otherwise the influence of the length of imprisonment would systematically bias the results. Nevertheless, comparison with a healthy control group would have been preferable. However, these data were not available to the level of detail required.

Matching enabled us to make a selection of cases from a previously representative dataset. Accordingly, the results cannot be transferred uncritically to remand or to female prisoners.
